# A protocol for a randomized controlled trial investigating the safety and cost-effectiveness of outpatient total hip arthroplasty

**DOI:** 10.1186/s12891-020-03699-z

**Published:** 2020-10-08

**Authors:** Bryn O. Zomar, Jacquelyn D. Marsh, Brent A. Lanting, Dianne M. Bryant

**Affiliations:** 1 Faculty of Health Sciences, University of Western Ontario, London, ON Canada; 2 Bone and Joint Institute, University of Western Ontario, London, ON Canada; 3 London Health Sciences Centre, London, ON Canada; 4 Schulich School of Medicine and Dentistry, University of Western Ontario, London, ON Canada

**Keywords:** Outpatient, Total hip arthroplasty, Same day discharge, Zelen, Cost-effect, Health economics, Safety, Cost

## Abstract

**Background:**

A significant proportion of the overall cost of total hip arthroplasty (THA) results from the inpatient hospital stay following the procedure. Considering the substantial and increasing number of these procedures performed annually, shifting to an outpatient model of care where the patient is discharged home the same day as their surgery represents a potential for significant cost savings. The potential significant impact of an outpatient care model on constrained healthcare budgets and lack of high-quality evidence regarding its effectiveness warrants a rigorous comparative trial. The purpose of this prospective, randomized controlled trial is to evaluate outpatient care pathways for THA. Specifically, our objectives are to compare the rate of serious adverse events and estimate the cost-effectiveness of outpatient compared to standard inpatient THA.

**Methods:**

We will include patients undergoing primary THA whom have an American Society of Anaesthetists status equal to or less than three, live within a 60-min driving distance of the institution and have an adult to accompany them home postoperatively and stay with them overnight. Consenting patients will be randomized to be discharged on the same day as surgery, as outpatients, or as inpatients according to standard of care (minimum of one night in hospital) using a modified Zelen consent model. The primary outcome measure is the incidence of serious adverse events at 30 days postoperative. Participants and their caregivers will complete secondary outcomes measures at each follow-up visit including patient-reported outcome measures and self-reported cost questionnaires.

**Discussion:**

This protocol is the first randomized trial to use blinding to evaluate outpatient THA compared to standard overnight stay and first to prospectively perform a full economic evaluation. It is also the first adequately powered trial to prospectively assess the safety of outpatient THA. Successful completion of this study could have the potential to provide clinical evidence for the role of outpatient THA in current practice.

**Trial registration:**

This study was retrospectively registered on ClinicalTrials.gov (NCT03026764) on March 9th, 2016.

## Background

Osteoarthritis (OA) is a leading cause of disability and reduced quality of life, presenting a substantial, growing burden to patients and the healthcare system [1]. Total hip arthroplasty (THA) is an established, effective intervention for advanced OA. The prevalence of hip OA is rapidly increasing, resulting in a rising demand for care. The number of THA procedures is projected to grow by 71% between 2014 and 2030 in the US [2]. Currently, THA, along with total knee arthroplasty (TKA), have a significant impact on healthcare budgets, costing approximately $1.2 billion in annual spending in Canada [3]. These staggering numbers highlight the critical need to improve care delivery.

A significant proportion of the overall cost of joint replacement results from the inpatient hospital stay following the procedure. Historically, the standard procedure following THA required an inpatient hospital stay of two and a half to 3 weeks, however the introduction of less invasive surgical techniques, improved medical and analgesia management and comprehensive rehabilitation have enabled shorter inpatient stays. Today, the median inpatient stay following THA is 3 days in Canada [3]. A desire for greater autonomy by the patients as well as patients wanting early mobilization to accelerate recovery and return to activities has led some clinicians to consider an outpatient arthroplasty program. The proposed benefits of outpatient arthroplasty include similar patient outcomes with significantly lower hospital costs, and improved patient satisfaction, independence, and autonomy. However, there is a lack of high-quality evidence comparing clinical outcomes of outpatient to inpatient arthroplasty models of care.

A retrospective analysis of over 50,000 THA and TKA procedures found no differences in 30-day major complications or readmissions among patients with a zero to two-day hospital stay compared to those discharged on day three or four postoperative [4]. Small cohort studies [5–8] suggest lower costs for outpatients and improved patient satisfaction but have inherent biases as they are limited to carefully selected patients in privatized health systems.

It is estimated that up to 20% of the overall cost of THA can be attributed to the inpatient stay in hospital at our institution [9]. By discharging patients as outpatients, it could be possible to have substantial savings. Although these preliminary calculations are encouraging, it is not sufficient to effect change solely to achieve cost control, without consideration of safety, effectiveness and patient satisfaction. Further, it is unknown whether the financial savings will be outweighed by additional postoperative costs, increased readmissions or decreased quality of care. A full economic evaluation that simultaneously evaluates cost and effectiveness is crucial prior to implementation. The lack of high-quality evidence regarding its effectiveness warrants a rigorous comparative trial.

The purpose of this study is to evaluate outpatient care pathways for THA. Specifically, our objectives are to compare the rate of serious adverse events and estimate the cost-effectiveness of outpatient compared to standard inpatient THA using a patient-blinded, randomized clinical trial.

## Methods/design

### Study setting

**Fig. 1:**
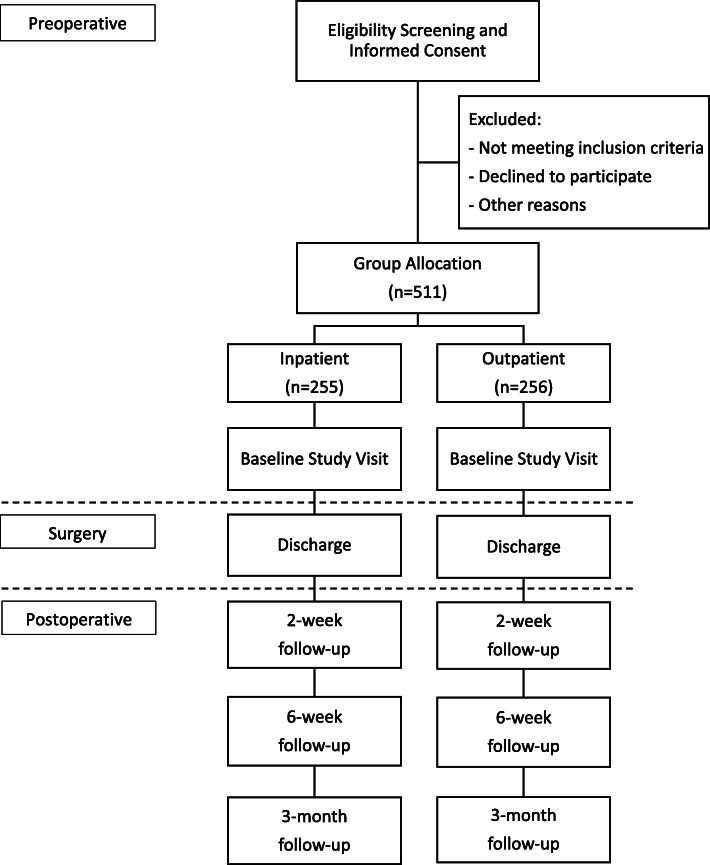
Participant flow through the study

Patients will be recruited from two orthopaedic centers in Canada (London Health Science’s University Hospital, London ON and St. Michael’s Hospital, Toronto ON). The surgeons will screen potential participants in their clinics and provide an overview of the research study (Fig. 1).

### Eligibility criteria

We will include patients undergoing primary THA. Eligible patients will have an American Society of Anesthetists (ASA) status equivalent or less than three, the ability to read and understand English, live within a 60 min commute distance of the institution, have access to a phone, and sufficient caregiver support. We will exclude patients with a history of anesthesia related complications, narcotic dependency, anaphylaxis to penicillin, significant psychosocial issues that influence safety, or cognitive issues that preclude the ability to understand instructions. We will also exclude patients whom have fibromyalgia, are skeletally immature, have an active or suspected latent infection in or about the joint, bone stock inadequate for support or fixation of the prosthesis, are unable to go to their home after surgery, have neuromotor conditions, significant pain management issues or obesity that significantly impacts their ability to mobilize (Table 1).

**Table 1:** Eligibility Criteria

Inclusion Criteria	Exclusion Criteria
1. Primary THA	1. Fibromyalgia
2. ASA status ≤3	2. Skeletally immature
3. Ability to read and understand English	3. Cognitive or neuromotor conditions
4. Live within 60-min commute distance of hospital	4. Bone stock inadequate for support or fixation of prosthesis
5. Home/cell phone access	5. Unable to go to their home after surgery
6. An adult to accompany patient home postoperatively	6. Active or suspected latent infection in or about the joint
	7. Significant pain management issues
	8. Patient/family history of anesthesia related complication(s)
	9. Obesity that significantly impacts the patient’s ability to mobilize
	10. Anaphylaxis to penicillin
	11. Significant psycho/social issues that would prevent the patient from managing at home safely
	12. Narcotic dependency

### Interventions

All patients will undergo a primary unilateral THA. All THA procedures will be performed using a direct anterior surgical approach according to the surgeon’s standard of care. The specific surgical pathway details (anesthetic, implants, etc.) will not be standardized for this study to maintain generalizability.

Both inpatient and outpatient care models begin with comprehensive preoperative patient education to equip the patient with a reasonable set of goals and expectations about their surgery, medications, pain management and rehabilitation. Immediately following surgery, the primary goals are to treat pain, nausea and hypovolemia. The combination of this early rehabilitation program, with a muscle sparing technique, is vital to the rapid recovery process. The surgical technique minimizes blood loss, provides a stable construct to facilitate immediate weightbearing, minimizes soft tissue trauma and includes a peri-articular injection. Home-based physical therapy should begin immediately, with a focus on ambulation.

Perioperative care and discharge protocols will be similar for both care models. Standard discharge criteria includes: ability to use required gait aids, appropriate pain control, control or absence of nausea and vomiting, hemostasis at the surgical wound, hemodynamically stable with appropriate laboratory values, alert and oriented, able to use the bathroom, meets the hospital standard targets from physiotherapy for discharge, given take-home medications, and in the company of a caregiver. Potential complications are also discussed so the patient understands the normal course of recovery as well as signs or symptoms that may be cause for concern and additional consultation.

#### Patients randomized to inpatient

Patients allocated to the inpatient group will be discharged home according to standard protocol at each site (typically one to 4 days postoperative).

#### Patients randomized to outpatient

Patients allocated to the outpatient group will be discharged home on the same day as the procedure. These patients will receive an additional physiotherapy session prior to surgery where they are instructed to practice certain skills such as bed transfer, cane/crutches use and gait training.

### Outcomes

We will collect outcome data preoperatively and at follow-up visits at discharge, 2 weeks, 6 weeks and 3 months postoperative, which is in keeping with usual practice patterns. We will record anaesthesia and surgical time and blood loss for each procedure and demographic information including date of birth, sex, height, weight, smoking status, and comorbidities.

#### Primary outcome

Given that the primary concern with sending patients home earlier than usual is an increase in the number or severity of early post-operative complications, our primary outcome is any serious adverse event within the first 30 days postoperative. A serious adverse event is defined as any untoward medical occurrence that results in death, is life-threatening, requires inpatient hospitalization or causes prolongation of existing hospitalization, results in persistent or significant disability or incapacity, or requires intervention to prevent permanent impairment or damage [10–12].

#### Secondary outcomes

Our secondary outcomes include patient-reported satisfaction, health-related quality of life (HRQOL), function, pain, and caregiver assistance. Patients will be asked to rate satisfaction with pain control, safety and quality of care on either a 100-mm visual analogue scale (VAS) with a score of 100 representing the highest possible satisfaction, or on a five to seven-point ordinal scale (completely satisfied to completely unsatisfied).

We will measure HRQOL using the *Western Ontario and McMaster Universities Osteoarthritis Index* (WOMAC), which includes three arthritis-specific domains of pain, stiffness and physical function. It has demonstrated content, cross-sectional and construct validity, good internal consistency reliability and moderate to high test retest reliability in patients undergoing THA and TKA [13]. To measure function, we will use the *Harris Hip Score* (HHS) [14]; a valid and reliable objective functional measure completed by the surgeon. We will measure general health using the *12-Item Short Form Health Survey* (SF-12v2) and th*e European Quality of Life Scale* (EQ-5D). The SF-12 evaluates limitations on physical and social activities, activities of daily living, pain, mental health and well-being, and perceptions of health and is valid, reliable, and responsive in patients with arthritis [15]. The EQ-5D index includes domains of mobility, self-care, usual activities, pain, anxiety and depression and has good test retest reliability and cross-sectional construct validity in patients with arthritis [16]. We will measure pain using a self-administered *Numeric Pain Rating Scale* (Pain NRS), ranging from 0 (no pain) to 10 (worst). We will measure the level of assistance provided by the caregiver throughout the recovery period using the *Caregiver Assistance Scale* (CAS) [17] and *Caregiver Strain Index* (CSI) [18].

#### Cost

We will ask patients to report any calls to the surgeon’s office, the on-call resident, the orthopaedic outpatient clinic, emergency room visits or hospitalizations for the first 2 weeks postoperative. Patients will also complete a healthcare resource use diary at each follow-up. The diary includes information on emergency room visits, hospitalizations, family doctor, specialist, healthcare professional or outpatient clinic visits, tests, procedures, and prescription or over-the-counter medications and any other miscellaneous costs related to their joint replacement. We will also record employment status and time-off paid employment, homemaking or volunteer activities, for both patients and caregivers.

We will obtain surgical procedure costs from our institutions’ case costing department. The unit costs for additional resource use following surgery can be found in provincial fee schedules and drug benefit formularies. The average Canadian wage reported by Statistics Canada will be applied to place a monetary figure on time off paid employment, for both patients and their caregivers and the current value of minimum wage in the province of Ontario to account for lost time for those who were retired, as well as time away from volunteer or home making activities.

The total cost will be determined by multiplying the quantity of resource use by the corresponding unit cost, summing the total cost over each follow up interval, and then calculating the mean cost at each follow-up time point, as well as an overall mean cost for the entire study period.

### Participant timeline

Patients will be screened and consent obtained at the time of booking for surgery and baseline assessments will occur at the preoperative visit within 3 months of surgery. On the day of discharge from hospital, we will obtain a pain score from participants and provide them with a daily diary to record costs for the first 2 weeks. Postoperatively, participants will be seen at 2 weeks, 6 weeks and 3 months for follow-up.

We will ask caregivers to complete questionnaires preoperatively and at 2 weeks postoperatively (Table 2).

**Table 2:** Participant Timeline

Assessments	Appointment				
	Baseline	Discharge	2 weeks	6 weeks	3 months
Demographics	X	–	–	–	–
Charlson Comorbidity Index	X	–	–	–	–
Self-Efficacy	X	–	–	–	–
Pain Catastrophizing Scale	X	–	–	–	–
Expectation Questionnaire: Preop	X	–	–	–	–
Surgical Information Form	–	X	–	–	–
Patient Flow	–	X	–	–	–
Pain NRS	X	X	X	X	X
SF-12v2	X	–	X	X	X
EQ-5D-5L	X	–	–	X	X
WOMAC	X	–	–	–	X
HHS	X	–	–	–	X
Adverse Event Form	–	X	X	X	X
Patient Satisfaction	–	–	X	–	–
Daily Diary	–	–	X	–	–
Patient Satisfaction and Expectations	–	–	X	X	X
Cost Questionnaire	–	–	X	X	X
Caregiver Demographics	X	–	–	–	–
Caregiver Assistance Scale	X	–	X	–	–
Caregiver Strain Index	X	–	X	–	–

### Recruitment

Patients presenting to the clinic will first be booked for THA by their surgeon. The surgeon will then assess whether the patient is eligible for possible outpatient discharge and will broadly discuss the study with the patient. The surgeon will then ask if the patient is interested in hearing more about the study and whether the research assistant may contact them with further information. Once patient approval is received, the patient is contacted by the research assistant with more information. All patients will be given the necessary time to consider their participation in the study and to provide informed consent if willing to participate.

### Randomization

#### Concealment mechanism/implementation

Potential participants will be initially screened by their treating surgeon and approached for participation by the research assistant. To reduce selection bias, patients are randomized into group using a stratified and blocked scheme after eligibility has been fully determined. The research assistant will confirm their eligibility and, after obtaining consent, will enter the patient’s date of birth and whether they’ve had previous experience with THA before randomizing the patient via a web-based randomization system to inpatient or outpatient.

#### Allocation (sequence generation)

All participants will be randomized by the research assistant using a 1:1 ratio for inpatient to outpatient. Randomization will be stratified by surgeon and previous experience with THA (had a THA on the contralateral limb or were the caregiver for someone whom had a THA). All patients will be analyzed according to the treatment group as assigned regardless of when they were discharged.

#### Blinding

To reduce detection bias, patients will be unaware that they were randomly assigned to a discharge plan and remain blinded to the presence of a comparison group until they reach the end of the study. Participants are kept blinded to group allocation using a modified Zelen consent model [19]. In this design, eligible patients are randomized to the intervention prior to providing consent to minimize the risk of bias associated with knowledge of the alternative intervention. We posit that patients with a bias for inpatient care who are randomized to the outpatient group may be more likely to return to seek additional care significantly biasing costs. Alternatively, patients with a strong preference for outpatient care who are randomized to the inpatient group may bias measures of satisfaction. Thus, patients are asked to consent to participation in a research study evaluating the outcomes and costs associated with patients undergoing THA; they are not told about randomization, the existence of an alternative group, or the between-groups objectives. At the end of the study, all patients are informed about the deception and asked to consent to having their data used for analysis.

As data collection occurs on the day of discharge from the hospital, it is not possible to blind the research assistant responsible for collecting patient-reported outcomes, however since outcomes are patient-reported, we do not expect an increase in the risk of detection bias. We are also unable to blind investigators to group allocation.

### Sample size

A retrospective analysis of over 50,000 THA and TKA procedures found no differences in 30-day major complications or readmissions among patients with a zero to two-day hospital stay compared to those discharged on day three or four postoperative [4]. The rate of serious adverse events in the inpatient group is expected to be greater than 5 % [20]. To define a non-inferiority margin we agreed that no more than a 5 % increase in the risk of serious adverse event (risk difference ≥ 6% favouring inpatient care) was acceptable. If the risk in the inpatient group ranges between five to 8 % and there is truly no difference in risk of serious adverse event between the groups, then a maximum of 506 patients are required to be 80% certain that the upper limit of a one-sided 95% confidence interval will exclude a difference in favour of the inpatient group of more than 6 % [21]. Given that the primary outcome is being measured at 30-days postoperative, we anticipate a low lost-to-follow-up rate. Previous studies conducted at the lead study site in similar patients demonstrate loss to follow up rates less than 1 % for the first 30 days. Therefore, to account for a potential 1 % dropout rate we inflated our sample size to 511.

### Plan for statistical analysis

We will present the incidence of serious adverse events by group at 30 days postoperative and calculate a risk difference and relative risk with 95% confidence intervals around the estimates. The mean and standard deviation for all continuous outcomes (Satisfaction, WOMAC, SF-12, HHS, Pain NRS, and CAS) will be calculated for each group at each time point and calculate the mean between-group difference with 95% confidence interval. Linear mixed models, using a covariance structure that allows for correlations between measurements to decline as they are further apart in time will be used to evaluate improvements in function after surgery since this provides a powerful approach to analyze complex longitudinal data while controlling for important covariates such as gender, comorbidities, age and surgeon/site. All data will be analyzed according to the intention-to-treat principle with an as treated analysis also performed.

We will conduct a cost-effectiveness analysis from both a Canadian healthcare payer and societal perspective using the incidence of adverse events as our effectiveness outcome to estimate cost-effectiveness at 3 months postoperative. We will determine cost-effectiveness using the net benefit regression (NBR) framework to estimate the incremental net benefit (INB) of outpatient arthroplasty [22]. An intervention is considered to be cost-effective if the INB is greater than zero. NBR also provides a means to adjust for potentially confounding factors and therefore allows greater statistical efficiency and a more precise estimate of the INB. To characterize the statistical uncertainty around our estimate of INB we will calculate 95% confidence intervals, and a cost-effectiveness acceptability curve [23].

### Patient and public involvement

Patients or the public were not involved in the design, or conduct, or reporting, or dissemination plans of our research.

### Data collection methods

Follow-up assessments for this study coincide with the standard of care follow-up schedule for the surgeons at our centre. During each visit, the research assistant will administer the questionnaires to the participants and ensure they are completed. Alternatively, participants are also given the option to complete questionnaires online by directly accessing the electronic data capture (EDC) system (*Empower Health Research*). The HHS will be completed by the treating surgeon at the three-month visit.

### Data management

Participants have the option to complete questionnaires either in hard copy or using the EDC system (*Empower Health Research*).

## Discussion

This protocol is for a multi-centered randomized controlled trial to assess the safety and cost-effectiveness of outpatient total hip arthroplasty compared to standard inpatient care. There is currently a lack of high-quality studies evaluating the safety and effectiveness of outpatient THA. Most studies that have assessed safety have used US national databases [24–29] or observational cohort designs [30–33] with only one published randomized trial [34]. Previous prospective studies have also been underpowered to detect differences in complication rates between outpatient and inpatient care groups. Due to the already low rates of serious adverse events after THA, reported from 3.8% up to 8.6% in the literature, [20, 35, 36] sample sizes must be sufficiently large to include a sufficient number of events to support clinical conclusions. Our protocol will be the first adequately powered prospective study to evaluate the safety of outpatient THA.

One strength of our study protocol is the use of a modified Zelen consent model [19], as one of the difficulties with assessing outpatient discharge in comparison with standard inpatient care is potential patient biases. This design allows us to avoid these biases as patients with a strong preference for either group are not made aware of the alternative intervention. A weakness of the randomized trial by Goyal et al. (2017) was the lack of blinding which led to a significant number of crossovers due to personal preference [34]. Our use of a modified Zelen consent model [19] should help to address this issue and reduce the number of crossovers in our study. Future studies looking to evaluate outpatient THA should also consider use of this design to strengthen their conclusions and reduce patient crossover.

Our study will be the first to perform a full economic evaluation in conjunction with a large randomized trial and will use both healthcare payer and societal perspectives. No full economic evaluations have been published to compare outpatient to standard inpatient care thus far. This is an important gap in the literature as international health economic guidelines suggest policy and clinical decisions should be supported by evidence produced when cost is evaluated simultaneously with effect in a full economic evaluation [37, 38]. Previous studies have evaluated cost alone [5, 6] and none in conjunction with a prospective trial. These studies have also only included direct costs associated with the procedures and hospital costs. Including a broader perspective and incorporating indirect costs such as time off work and caregiver assistance are important to consider, ensuring that costs possibly saved from reduced hospital stays are not shifted elsewhere in the recovery pathway. To truly know whether outpatient THA is less costly than inpatient care, as the literature currently suggests [5, 6], broader perspectives (ie. healthcare payer and societal) should be assessed. The use of multiple perspectives also helps to broaden the interpretability of study results and is recommended by international health economic guidelines [37]. The importance of costing perspective is highlighted in a paper by Primeau et al. (2019) where the conclusions of the study changed depending on the perspective used [39].

Our protocol hopes to address several gaps in the current literature with a large and adequately powered sample size to assess safety, the use of blinding with a modified Zelen consent model [19], and assessing costs in conjunction with a prospective randomized trial for a full economic evaluation. Conclusions from our study should help to inform clinical decision making in the use of outpatient care programs in standard practice for THA. Recruitment for the study is currently ongoing.

## Data Availability

Not applicable.

## Abbreviations

ASA: American Society of Anesthetists
CAS: Caregiver Assistance Scale
CSI: Caregiver Strain Index
EDC: Electronic data capture
EQ-5D: European Quality of Life Scale
HHS: Harris Hip Score
HRQOL: Health-related quality of life
INB: Incremental net benefit
NBR: Net benefit regression
OA: Osteoarthritis
Pain NRS: Numeric Pain Rating Scale
SF-12v2: 12-Item Short Form Health Survey
THA: Total hip arthroplasty
TKA: Total knee arthroplasty
VAS: Visual analog scale
WOMAC: Western Ontario and McMaster Universities Osteoarthritis Index
